# Phase 2, double-blind, randomized, placebo-controlled study of the safety and efficacy of elagolix in women with polycystic ovary syndrome

**DOI:** 10.1016/j.xfre.2023.02.007

**Published:** 2023-02-21

**Authors:** Michael C. Snabes, Juki Ng, Hong Li, Izna Ali, Mohamad Shebley, William D. Schlaff

**Affiliations:** a AbbVie Global, LLC, North Chicago, Illinois; b Jefferson Obstetrics and Gynecology, Philadelphia, Pennsylvania

**Keywords:** Polycystic ovarian syndrome, elagolix, menstrual cycle, ovulation, GnRH antagonist

## Abstract

**Objective:**

Evaluate the efficacy and safety of elagolix, a GnRH antagonist, to treat polycystic ovarian syndrome (PCOS).

**Design:**

A phase 2, multicenter, double-blind, randomized, placebo-controlled trial.

**Setting:**

Outpatient and academic medical centers.

**Patient(s):**

One hundred fourteen women with PCOS (aged 18–35 years, body mass index 18.5–38 kg/m^2^).

**Intervention(s):**

Patients were randomized 2:2:2:2:2:3 to elagolix (25 mg twice daily, 50 mg once daily, 75 mg twice daily, 150 mg once daily, and 300 mg twice daily) or placebo.

**Main Outcome Measure(s):**

The primary endpoint was menstrual cycle normalization (defined as 2 menstrual cycles 21–35 days in length during the 4-month treatment period). The secondary endpoint was change from baseline to week 1 in the area under the luteinizing hormone (LH) serum concentration-time curve (AUC). Additional endpoints included change from baseline in serum hormone levels.

**Result(s):**

No significant improvement in restoring normal menstrual cycles was observed in treated subjects; 3 of 114 patients met the primary endpoint. Six patients experienced progesterone elevations indicative of ovulation. The LH levels decreased from baseline to week 16, and LH AUC was significantly reduced from baseline to week 1 in all elagolix treatment groups (*P*<.1 vs placebo). Follicle-stimulating hormone (FSH) levels generally remained stable through week 16, with no significant differences in FSH AUCs. Serum estradiol and testosterone concentrations were consistently reduced from baseline in all elagolix dose groups compared with placebo. Adverse event rates were similar across treatment groups.

**Conclusion(s):**

Elagolix treatment did not normalize the ovulatory cycle in patients with PCOS.

**Clinical Trial Registration Number:**

NCT03951077

Polycystic ovary syndrome (PCOS) is one of the most common endocrine disorders in women, and, depending on the diagnostic criteria used, PCOS has an estimated prevalence of between 7% to 17% of reproductive-aged women (1, 2). Present treatment options are directed by the clinical goals of the affected patients. Those trying to conceive are treated with ovulation-inducing medications, whereas those who are not pursuing pregnancy generally seek treatments to address specific symptoms such as irregular and unpredictable periods or hirsutism. Most women with PCOS, regardless of their reproductive status, are concerned over health risks, including insulin resistance and glucose intolerance, heart disease, endometrial hyperplasia, and endometrial cancer, among others.

The diagnosis of PCOS requires the presence of at least 2 of 3 diagnostic criteria, namely oligomenorrhea (which is generally associated with chronic anovulation), polycystic morphology as seen on ultrasound, and biochemical or clinical hyperandrogenism; the latter generally manifests as hirsutism, acne, or androgenic alopecia (3). Additional findings associated with PCOS include, among others, obesity, insulin resistance, or gonadotrophic abnormalities (3).

Although the pathophysiology of PCOS is not yet fully understood, it is known to be based on dysfunction in the hypothalamic-pituitary-ovarian axis (4), and a great majority (approximately 87%) of patients have functional ovarian hyperandrogenism (5). Two arcuate nucleus populations are thought to be of interest in the etiopathogenesis of PCOS: the kisspeptin/neurokinin B/dynorphin-expressing “KNDy” neurons and the arcuate nucleus GABAergic neurons (6, 7, 8). The KNDy neurons are responsible for the pulse frequency of gonadotrophin-releasing hormone (GnRH) released from the arcuate nucleus that regulates the secretion of luteinizing hormone (LH) and follicle-stimulating hormone (FSH) from the pituitary gonadotrophs (6). Circulating LH levels are usually tonically elevated in PCOS compared with the LH levels in normal ovulatory cycles (9, 10, 11). Conversely, FSH levels are usually equal to or lower than those in women without PCOS (3, 10, 11). We note that classic studies in rhesus monkeys with median eminence lesions have shown that changes in the frequency of GnRH pulses that are presented to the pituitary alter the ratio of LH and FSH secreted systemically (12).

Elagolix is an orally administered, short-acting, nonpeptide, small-molecule GnRH antagonist that is indicated for the treatment of moderate to severe pain associated with endometriosis and heavy menstrual bleeding associated with uterine fibroids (13). In both human and nonhuman studies, elagolix has demonstrated a virtually immediate, dose-dependent, and highly significant reduction in LH and sex steroid levels (14, 15). Elagolix treatment also reduces circulating FSH levels in a dose-dependent manner, but to a lesser extent than LH suppression (15, 16). Results from studies of other GnRH antagonists, such as cetrorelix and ganirelix, have also demonstrated greater suppression of LH than FSH levels with GnRH inhibition (15, 17).

We hypothesized that an optimal dose and regimen of elagolix might be identified which would be insufficient to completely suppress the hypothalamic-pituitary-ovarian axis but would be adequate to reduce LH secretion while maintaining circulating FSH and possibly lead to a reinitiation of follicular development. Were this hypothesis confirmed, we would postulate that we might be able to implement this approach to restore normal follicular dynamics and androgen levels and hopefully produce predictable ovulation and regular menses in patients with PCOS.

## Materials and methods

### Trial Design

This 6-month, phase 2, randomized, double-blind, placebo-controlled, parallel-group, multicenter study was performed to assess the safety and efficacy of elagolix in reducing LH and androgen levels and producing regular menses in women with PCOS. This trial was conducted between September 20, 2019, and February 10, 2021, across 35 study sites (outpatient and academic medical centers) in the United States and Puerto Rico. All study sites, investigators, patients, and data reviewers were blinded for the duration of the study. The study was conducted per the protocol; International Council for Harmonization guidelines; and applicable regulations, guidelines, and ethical principles originating from the Declaration of Helsinki. All protocols were approved by an institutional review board (Advarra Inc., Columbia, MD, USA), and patients provided written informed consent before screening or undergoing study-specific procedures. The study was registered at ClinicalTrials.gov (NCT03951077).

Study eligibility criteria included women aged 18 to 35 years with a body mass index (BMI) of 18.5 to 38.0 kg/m^2^ and a diagnosis of PCOS. Other inclusion criteria included FSH levels ≤10 IU/L and elevated total or free testosterone levels. Women were excluded from the study if there was a history of other conditions resulting in irregular menstrual bleeding, such as uterine polyps or fibroids; unstable/untreated medical conditions or those requiring intervention during the study; laser hair removal within the previous 6 months; history of alcohol/drug abuse or suicide attempts; clinically significant gynecological findings on imaging; endocrine diseases such as Cushing’s syndrome and diabetes mellitus; osteoporosis or another metabolic bone disease; or current pregnancy, current breastfeeding, or pregnancy within the previous 6 months.

The screening took place over 6 weeks and included evaluation of the above eligibility criteria, initiation of e-Diary entries, and measurement of baseline endocrine hormone levels. After the screening period, eligible patients were randomized based on a 2:2:2:2:2:3 ratio (active treatment to placebo) using an interactive response technology with stratified randomization according to baseline Ferriman–Gallwey score (<8, ≥8) and baseline BMI (<30, ≥30). The elagolix groups received 25 mg twice daily, 50 mg once daily, 75 mg twice daily, 150 mg once daily, and 300 mg once daily. The treatment intervention was planned to be performed over 24 weeks, and patients were evaluated with weekly progesterone blood draws. After completion of treatment, a 30-day after treatment follow-up was planned.

Serial blood samples were taken from patients for LH and FSH assays at baseline and at weeks 1, 4, and 16. The LH and FSH sparse sampling were performed at weeks 8, 12, 20, 24, and at the 30-day follow-up. Serum testosterone and estradiol levels were evaluated at baseline and weeks 4, 8, 12, 16, 20, 24, and at the 30-day follow-up. Progesterone levels were measured weekly; a level ≥3 ng/mL was considered indicative of ovulation. Additionally, patients were to maintain a daily bleeding diary throughout the screening and treatment period; “bleeding” was defined as 1 or more tampons or pads required within 24 hours.

### Endpoints

The primary endpoint was the proportion of menstrual cycle responders. A patient was considered a menstrual cycle responder if she had at least 2 menstrual cycles during the 4-month treatment period; a menstrual cycle was defined as menstrual bleeding with a cycle length of 21 to 35 days (at least 2 consecutive days of bleeding or at least 1 day of bleeding immediately followed by 1 day of spotting, or at least 1 day of spotting immediately followed by 1 day of bleeding, excluding spotting only). The secondary endpoint was the change from baseline to week 1 in the area under the LH serum concentration-time curve (AUC). Additional relevant endpoints included mean circulating LH, FSH, estradiol, and testosterone levels during the treatment period and change from baseline in the LH serum AUC and FSH serum AUC at weeks 1, 4, and 16.

Safety evaluations included physical examinations, vital sign assessments, laboratory parameter measurements, imaging, bone mineral density measurements, and adverse event (AE) monitoring for the entire duration of the study. Treatment-emergent AEs were defined as events beginning after the first dose but also occurring within 30 days after the last dose of the study drug. The AEs were coded according to the system organ class and preferred term of the Medical Dictionary for Regulatory Activities version 23.1.

### Statistical Analysis

The planned sample size was 130 patients (20 per elagolix treatment group and 30 assigned to the placebo group), which would provide at least 85% power to detect a difference between each elagolix group and the placebo group in the proportion of menstrual cycle responders, assuming response rates of 10% for the placebo group and 50% for the elagolix group with 2-sided α = 0.05. This sample size was calculated using nQuery adviser 7.0. We compared each elagolix group to the placebo group in the primary efficacy endpoint analysis using statistical analyses at a 2-sided alpha level of 0.1. The difference in responder proportions between the treatment groups and the placebo group was analyzed using the Cochran-Mantel-Haenszel test adjusted for Ferriman–Gallwey score (<8, ≥8) and BMI (<30, ≥30) at baseline. In addition, the Cochran-Mantel-Haenszel–based 95% confidence interval (95% CI) for the difference in the proportion of responders within each Ferriman–Gallwey score and BMI at baseline was calculated based on normal approximation.

The analysis for the key secondary endpoint was based on a mixed-effects model with repeated measures. The mixed-effects model with repeated measures analysis included the fixed categorical effects of treatment, visit, treatment-by-visit interaction, baseline Ferriman–Gallwey score (<8, ≥8), and baseline normal/obese status (BMI <30, ≥30); subject as a random effect; and baseline measurement. Each elagolix group was compared with the placebo group using the least squares mean of treatment difference with a 90% CI; associated *P* values were reported.

## Results

### Patients

**Supplementary Figure 1:**
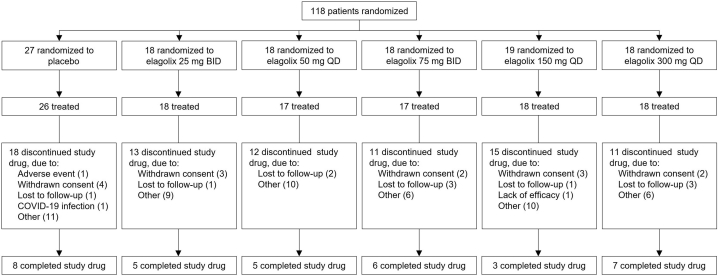
Patient disposition.

After an interim analysis, the study was prematurely terminated as it failed to meet the primary endpoint. Although the number of participants planned for the study was 130, only 118 were randomized, of which 114 patients received at least 1 dose of the study drug and 34 completed the 6-month treatment period (Supplementary Figure 1, available online). Patient demographics and baseline characteristics were similar across the treatment groups (Table 1). A total of 80 patients discontinued the study drug (elagolix 25 mg twice daily, n = 13; elagolix 50 mg once daily, n = 12; elagolix 75 mg twice daily, n = 11; elagolix 150 mg once daily, n = 15; elagolix 300 mg once daily, n = 11; placebo, n = 18); the primary reasons for study drug discontinuation overall included withdrawn consent (n = 14), lost to follow-up (n = 11), AE (n = 1, placebo group), lack of efficacy (n = 1), COVID-19 infection (n = 1), or other (n = 52).

**Table 1:** Baseline characteristics and patient demographics.

Parameter	Placebo (n = 26)	Elagolix 25 mg BID (n = 18)	Elagolix 50 mg QD (n = 17)	Elagolix 75 mg BID (n = 17)	Elagolix 150 mg QD (n = 17)	Elagolix 300 mg QD (n = 18)	Total (N = 114)
Age (y)	27.8 (4.6)	25.3 (5.2)	27.6 (4.9)	26.9 (3.3)	25.1 (4.2)	26.1 (4.4)	26.5 (4.5)
Race							
White, n (%)	19 (73.1)	13 (72.2)	16 (94.1)	12 (70.6)	11 (61.1)	14 (77.8)	85 (74.6)
Black, n (%)	6 (23.1)	4 (22.2)	1 (5.9)	5 (29.4)	7 (38.9)	4 (22.2)	27 (23.7)
Other, n (%)	1 (3.8)	1 (5.6)	–	–	–	–	2 (1.8)
Ethnicity, not Hispanic/Latino, n (%)	21 (80.8)	13 (72.2)	11 (64.7)	14 (82.4)	14 (77.8)	14 (77.8)	87 (76.3)
Weight, kg	83.8 (13.0)	89.3 (14.0)	86.5 (14.2)	87.2 (15.0)	86.4 (16.1)	87.4 (12.5)	86.6 (13.9)
BMI, kg/m^2^, n (%)	31.3 (4.8)	34.4 (5.0)	32.3 (5.2)	32.1 (4.5)	32.6 (4.2)	32.6 (4.4)	32.5 (4.7)
Smoker							
Current, n (%)	5 (19.2)	1 (5.6)	2 (11.8)	5 (29.4)	5 (27.8)	1 (5.6)	19 (16.7)
Former, n (%)	3 (11.5)	2 (11.1)	–	1 (5.9)	1 (5.6)	2 (11.1)	9 (7.9)
Never, n (%)	18 (69.2)	15 (83.3)	15 (88.2)	11 (64.7)	12 (66.7)	15 (83.3)	86 (75.4)

*Note:* All data are presented as mean ± standard deviation unless otherwise indicated.

BID = twice daily; BMI = body mass index; QD = once daily; y = year.

### Primary Endpoint

Out of 114 participants, only 3 (2.6%) met the primary endpoint of menstrual cycle regulation: 2 in the placebo group and 1 in the elagolix 300-mg group. There were no statistical differences observed between the placebo group and any of the elagolix treatment groups for the primary endpoint. Six patients experienced elevated progesterone levels (> 3 ng/mL) on more than 1 occasion during the treatment period—4 patients who received elagolix and 2 who received a placebo.

### Hormonal Analyses

**Figure 1:**
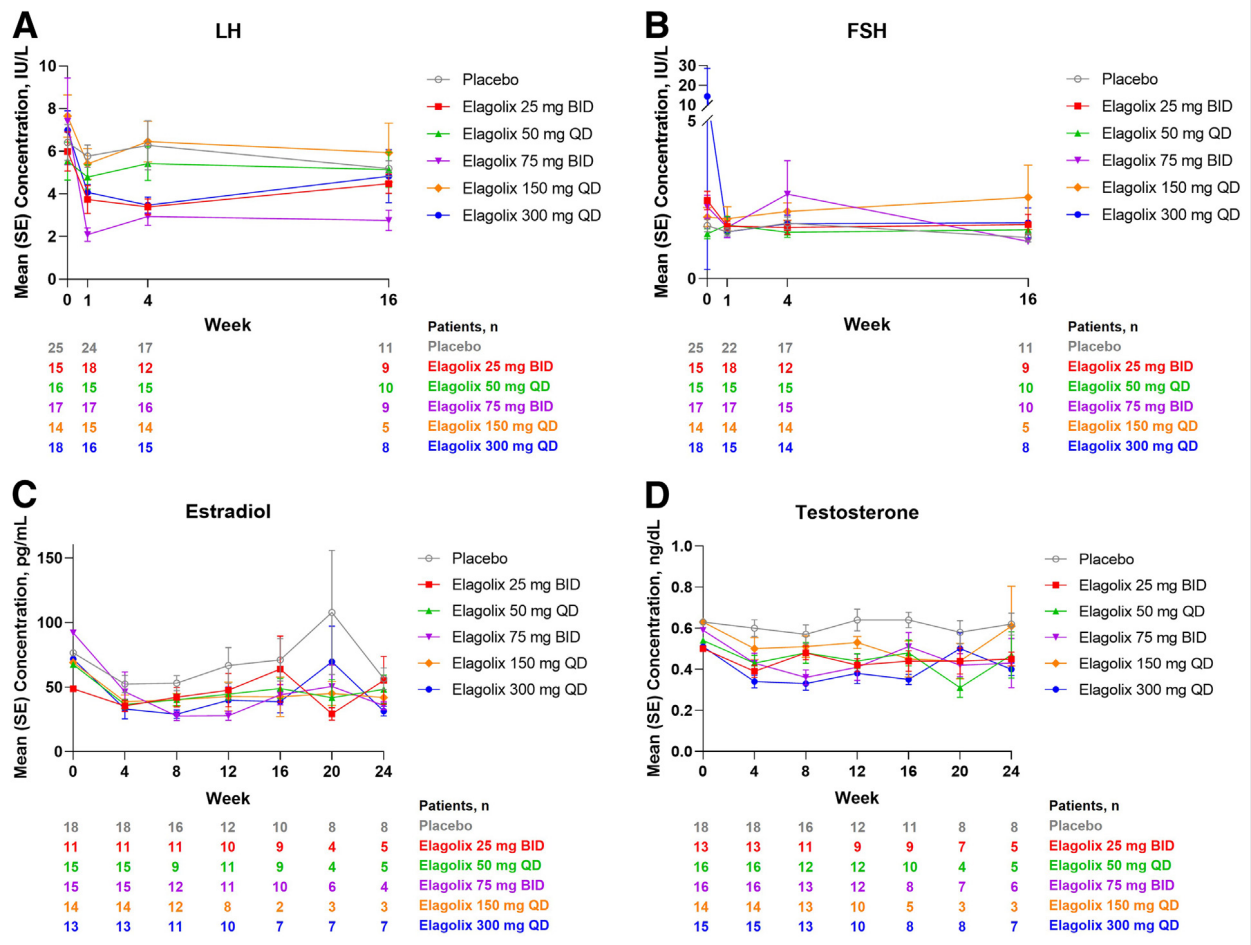
Mean luteinizing hormone (A), follicle-stimulating hormone (B), estradiol (C), and testosterone (D) levels at each visit over time. BID = twice daily; FSH = follicle-stimulating hormone; IU/L = international units per liter; LH = luteinizing hormone; QD = once daily; SE = standard error.

Analysis of LH levels revealed a decrease from baseline to week 4 with elagolix treatment that was generally maintained through week 16 (Figure 1A). A statistically significant decrease from baseline in LH AUC between each elagolix group and placebo was observed at week 1 (*P*<.1; Table 2); the reduction in LH AUC was also observed through week 4. A dose-dependent reduction in LH AUC was not apparent. As the study was discontinued early and treatment was stopped for all study participants, the amount of hormone data available in week 16 is much smaller to allow meaningful comparison.

**Table 2:** Mean change from baseline in LH and FSH AUCs.

AUC	Placebo	Elagolix 25 mg BID	Elagolix 50 mg QD	Elagolix 75 mg BID	Elagolix 150 mg QD	Elagolix 300 mg QD
LH						
Baseline mean (n)	26.1 (24)	21.5 (15)	20.6 (15)	25.6 (17)	30.0 (13)	27.8 (16)
Change from baseline, LS mean (SE) [n, *P* value]						
Week 1	−1.8 (2.3) [24, —]	−7.8 (2.9) [15, .087^a^]	−10.7 (2.9) [15, .012^a^]	−17.9 (2.7) [16, <.001^a^]	−8.0 (3.1) [13, .091^a^]	−13.5 (2.8) [15, <.001^a^]
Week 4	0.8 (2.5) [17, —]	−12.7 (3.2) [10, <.001^a^]	−7.1 (2.8) [14, .027^a^]	−14.7 (2.5) [16, <.001^a^]	−4.8 (3.0) [12, .132]	−13.3 (2.7) [14, <.001^a^]
Week 16	−5.8 (2.6) [10, —]	−8.1 (3.1) [7, .568]	−8.9 (2.8) [10, .400]	−14.7 (2.6) [10, .017^a^]	−7.1 (4.2) [4, .795]	−8.7 (3.2) [7, .472]
FSH						
Baseline mean (n)	401.2 (23)	516.1 (15)	337.2 (14)	514.2 (17)	473.5 (12)	425.2 (16)
Change from baseline, mean (SD) [n, *P* value]						
Week 1	−37.0 (43.4) [23, —]	−51.8 (53.4) [15, .815]	2.4 (54.2) [14, .536]	−71.7 (48.0) [17, .570]	−9.9 (58.9) [12, .686]	−98.0 (48.6) [16, .321]
Week 4	−19.7 (69.6) [17, —]	−189.6 (88.5) [10, .123]	6.8 (80.8) [14, .797]	33.4 (74.4) [16, .595]	−6.4 (87.5) [12, .902]	−44.2 (75.5) [15, .808]
Week 16	−89.8 (58.8) [9, —]	−94.2 (67.1) [7, .960]	−50.4 (59.1) [10, .620]	−227.1 (55.7) [10, .093^a^]	35.9 (89.7) [4, .238]	−28.6 (62.8) [8, .466]

*Note: P* values were calculated using MMRM with the fixed categorical effects of treatment, visit, treatment-by-visit interaction, baseline FG score (<8, ≥8), and baseline normal/obese status (BMI <30, ≥30), subject as a random effect, and the continuous fixed covariate of baseline measurement.

AUC = area under the serum concentration-time curve; BID = twice daily; BMI = body mass index; FG = Ferriman–Gallwey; FSH = follicle-stimulating hormone; LH = luteinizing hormone; MMRM = mixed-effect model repeat measurement; QD = once daily; SE = standard error.

a *P*<.1 vs placebo.

Unlike the reduction in LH levels, analysis of FSH concentrations demonstrated that FSH levels were generally maintained through 16 weeks of elagolix treatment (Figure 1B). FSH AUCs were also not significantly affected by elagolix treatment compared with placebo treatment (*P*≥.321 at week 1 and *P* ≥.123 at week 4 for all elagolix groups; Table 2).

Serum estradiol concentrations decreased in all treatment groups from baseline through week 24 (Figure 1C). Estradiol appeared to be suppressed over time in most elagolix groups compared with placebo, and there was no evidence of dose-dependent reductions (Table 3). Additionally, there was no trend of increased serum estradiol for any specific elagolix dose.

**Table 3:** Mean change from baseline in estradiol and testosterone

Parameter	Placebo	Elagolix 25 mg BID	Elagolix 50 mg QD	Elagolix 75 mg BID	Elagolix 150 mg QD	Elagolix 300 mg QD
Estradiol, pg/mL						
Baseline mean (n)	76.5 (18)	48.7 (11)	67.4 (15)	92.1 (15)	69.1 (14)	71.7 (13)
Change from baseline, LS mean (SE) [n]						
Week 4	−21.8 (9.5) [18]	−35.5 (11.7) [11]	−36.6 (10.3) [15]	−30.1 (10.0) [15]	−35.3 (10.9) [14]	−39.9 (10.7) [13]
Week 8	−20.1 (10.0) [16]	−27.7 (11.7) [11]	−33.5 (13.0) [9]	−46.0 (11.0) [12]^a^	−33.7 (11.6) [12]	−45.0 (11.6) [11]^a^
Week 12	−5.7 (11.2) [12]	−21.2 (12.2) [10]	−28.7 (11.8) [11]	−44.9 (11.4) [11]^a^	−29.7 (13.7) [8]	−32.6 (12.1) [10]^a^
Week 16	−5.9 (12.1) [10]	−5.0 (12.7) [9]	−24.5 (12.9) [9]	−28.5 (11.9) [10]	−30.0 (25.4) [2]	−34.8 (14.1) [7]
Week 20	36.1 (13.2) [8]	−28.7 (18.1) [4]^a^	−30.2 (18.4) [4]^a^	−25.8 (15.1) [6]^a^	−26.7 (21.1) [3]^a^	−4.1 (14.2) [7]^a^
Week 24	−15.1 (13.2) [8]	−4.4 (16.4) [5]	−24.0 (16.6) [5]	−34.7 (18.0) [4]	−29.9 (21.1) [3]	−41.3 (14.1) [7]
Testosterone, ng/dL						
Baseline mean (n)	0.63 (18)	0.50 (13)	0.54 (16)	0.59 (16)	0.63 (14)	0.51 (15)
Change from baseline, LS mean (SE) [n]						
Week 4	0.01 (0.03) [18]	−0.15 (0.04) [13]^a^	−0.12 (0.04) [16]^a^	−0.14 (0.03) [16]^a^	−0.09 (0.04) [14]^a^	−0.19 (0.04) [15]^a^
Week 8	−0.01 (0.4) [16]	−0.05 (0.04) [11]	−0.06 (0.04) [12]	−0.20 (0.04) [13]^a^	−0.08 (0.4) [13]	−0.20 (0.04) [13]^a^
Week 12	0.02 (0.04) [12]	−0.06 (0.04) [9]	−0.10 (0.04) [12]^a^	−0.14 (0.04) [12]^a^	−0.06 (0.04) [10]	−0.14 (0.04) [10]^a^
Week 16	0.04 (0.04) [11]	−0.06 (0.04) [9]^a^	−0.05 (0.04) [10]	−0.08 (0.05) [8]^a^	−0.13 (0.06) [5]^a^	−0.19 (0.05) [8]^a^
Week 20	−0.01 (0.05) [8]	−0.07 (0.05) [7]	−0.12 (0.06) [4]	−0.16 (0.05) [7]^a^	−0.16 (0.07) [3]^a^	−0.04 (0.05) [8]
Week 24	0.03 (0.05) [8]	−0.03 (0.06) [5]	−0.01 (0.06) [5]	−0.13 (0.05) [6]^a^	0.01 (0.07) [3]	−0.13 (0.05) [7]^a^

*P* values were calculated using MMRM with the fixed categorical effects of treatment, visit, treatment-by-visit interaction, baseline FG score (< 8, ≥8), and baseline normal/obese status (BMI <30, ≥30), subject as a random effect, and the continuous fixed covariate of baseline measurement.

BID = twice daily; BMI = body mass index; FG, Ferriman–Gallwey; LS = least squares; MMRM = mixed-effect model repeat measurement; QD = once daily; SE = standard error.

a *P*<.1 vs placebo.

Testosterone levels also decreased with elagolix treatment (Figure 1D). As early as week 4, all groups had statistically significantly decreased mean testosterone values vs placebo, and these decreases were generally maintained over 24 weeks in most elagolix groups; dose-dependent reductions were not observed **(**Table 3).

### Safety

The AE rates were similar among elagolix groups compared with placebo. The most frequently reported AEs in the elagolix groups (N = 88) were COVID-19 (5.7%), urinary tract infection (4.5%), anemia (3.4%), hot flush (3.4%), nausea (1.1%), and arthralgia (1.1%). One serious AE of severe chronic cholecystitis occurred in the elagolix 25 mg twice daily group but was assessed and deemed by the investigator to be unrelated to the drug and did not lead to study drug discontinuation. Severe AEs occurred in 3 patients; all other AEs were considered by the investigator to be mild or moderate in severity. Investigators categorized all 4 severe AEs (Covid-19, anemia, back pain, and chronic cholecystitis) that occurred in these 3 patients as having "no reasonable possibility" of being related to the study drug. No deaths were reported at any time during the study.

## Discussion

The primary endpoint of menstrual cycle regulation in women with PCOS was not met in any of the study groups, and, as a result, this study was terminated early. There was a low frequency of AEs reported in the elagolix treatment groups, and the AEs reported in this study were largely consistent with those reported in previous elagolix monotherapy studies (18, 19, 20).

The objective of this study was to determine if an optimal dose of elagolix could be identified that would reduce LH levels while maintaining FSH levels without suppressing the hypothalamic-pituitary-ovarian axis. Early work from Wildt et al demonstrated that decreased frequency of GnRH pulses presenting to the pituitary of monkeys with median eminence lesions led to a decrease in serum LH but increased circulating FSH levels (12). Based on these findings from Wildt et al (12), we hypothesized that decreased LH (but not FSH) levels with GnRH antagonist treatment might result in the progression of follicular development with an increase in circulating estradiol and, potentially, LH surges, ovulation, and a normal luteal phase. We did not observe increased FSH levels nor any reinitiation of follicular development based on the uniform inhibition of estradiol levels observed. Our sampling protocol may not enable the detection of LH surges, but very few subjects exhibited ovulatory progesterone levels supporting that ovulation had occurred. Although we observed reduced LH levels with minimal effects on FSH levels, very few patients met the primary endpoint of menstrual cycle regulation. Furthermore, all doses of elagolix in this study decreased estradiol and testosterone levels, suggesting that all doses of elagolix were inhibitory to follicular development. Taken together, these results suggest that elagolix treatment by itself may not be a suitable treatment for patients with PCOS with respect to reinitiating follicular development. These findings are supported by other studies that reported suppression of LH and FSH levels with GnRH antagonists in healthy women (21, 22) and demonstrated reductions in circulating LH, FSH, and estradiol levels after GnRH antagonist treatment in patients with PCOS (23).

Our findings may be challenging to clearly interpret, given the limited amount of hormonal data collected and the fact that hormones such as LH, testosterone, and estradiol normally undergo constant fluctuations, even in nonovulatory women. However, the trends we observed may have interesting implications for future studies. All doses evaluated in this study had similar effects on the hormonal levels measured, suggesting the possibility that a lower dose of elagolix or a different dosing regimen for patients with PCOS could reduce LH tone a bit less profoundly and lead to the clinical goals set for this study. To better evaluate this possibility, a future study might need more frequent sampling, possibly supplemented by ultrasound assessment of folliculogenesis. An alternative approach could evaluate a low-dose GnRH antagonist (ie, lower than the doses evaluated in the current study) in conjunction with clomiphene or letrozole to potentially increase the chance of ovulation induction in patients with PCOS who are seeking fertility. This approach could be used optimally in patients with PCOS who do not respond predictably or at all to typical regimens used to induce ovulation. If these or other future studies are successful in reversing the LH/FSH ratio and reinitiating follicular development, findings could be applied for potential benefits in insulin resistance, endometrial hyperplasia, hair growth, acne, etc. We are not certain that insulin resistance would be alleviated if our hypothesis had been met, but it is possible that normalization of the steroid milieu over a long period could affect insulin sensitivity and potentially impact BMI.

Limitations of this study include small sample sizes in each treatment group, which substantially decreased over the course of the study because the study was discontinued early and treatment was stopped for all patients, limiting the amount of data available at later time points. Additionally, although LH, estradiol, and testosterone levels were generally suppressed with elagolix treatment, the large variability observed in hormone levels may have contributed to the lack of statistically significant differences between the placebo and elagolix groups. These findings are not generalizable to other patients with PCOS because of the small patient numbers and early trial discontinuation. Finally, menstrual cycles are irregular in patients with PCOS and may occur spontaneously, regardless of treatment, which poses a challenge to interpreting the effect of elagolix treatment on menstrual cycle regulation.

## Conclusion

In this phase 2 study evaluating the efficacy and safety of elagolix for the treatment of PCOS, normalization of the ovulatory cycle was not observed, suggesting that elagolix alone may not be a suitable treatment for PCOS.
